# Diphtheria

**Published:** 1873-02

**Authors:** 


### DIPHTHERIA.

In the earliest stages, when there is only soreness and swelling in the throat, gargle the throat every ten minutes with strong salt water; at the same time dip three folds of flannel in boiling salt water, press it out between folds of a towel, so as not to dribble, apply it to the throat as hot as the patient can bear it, covering it with a dry and larger flannel, so as to keep the hot flannel in close contact with the skin; renew this at each gargling, sprinkling fine salt on the side of the successive flannels which will touch the skin. But always remember that the sense of prostration is so insufferable, that the system must be supported every hour with beef tea, or other form of stimulant. The two symptoms always present in diphtheria are a very offensive breath, and dreadful debility.

